# MOMENTARY SOLITUDE AND WELL-BEING ACROSS ADULTHOOD: THE MODERATING ROLE OF TRAIT MINDFULNESS

**DOI:** 10.1093/geroni/igae098.3504

**Published:** 2024-12-31

**Authors:** Xinyuan Peng, Da Jiang, Dwight Tse, Yuen Wan Ho, Jennifer Lay

**Affiliations:** The Education University of Hong Kong, Hong Kong, China (People’s Republic); The Education University of Hong Kong, Hong Kong, China (People’s Republic); University of Strathclyde, Glasgow, Scotland, United Kingdom; Lingnan University, Hong Kong, Guangdong, China; University of Exeter, Exeter, England, United Kingdom

## Abstract

Solitude (an objective state of being alone without social contact) evokes positive and negative experiences. People spend more time alone as they age. Little is known about factors influencing affective experiences in solitude. Trait mindfulness facilitates emotion regulation in various social contexts. The person-environment fit theory posits that individuals enjoy better well-being in environments aligning with their traits. Mindfulness emphasizes self-awareness and stress reduction, which seems consistent with the nature of solitude. Thus, we aimed to investigate whether trait mindfulness would moderate the relationship between solitude and affective well-being in a lifespan sample. An experience sampling study (N = 191; Mage = 51.49, SDage = 17.70, range = 19-93, 66% women) was conducted. Participants completed smartphone-based assessments measuring momentary social situations (in solitude vs. not) and affective experiences five times per day for seven consecutive days. High- and low-arousal positive (PA) and negative affect (NA) were assessed with nine items. Five facets of trait mindfulness were examined as moderators. Results revealed that mindfulness facets played protective roles in maintaining well-being in solitude. Higher levels of “describing” and “acting with awareness” weakened the negative association between solitude and high-arousal PA. A higher level of “nonreactivity to inner experiences” amplified the positive association between solitude and low-arousal PA. A higher level of “observing” weakened the positive association between solitude and low-arousal NA. Age did not moderate these relationships. These findings enhance understanding of affective well-being in solitude across adulthood by highlighting the roles of different facets of mindfulness.

